# Immune compartments at the brain’s borders in health and neurovascular diseases

**DOI:** 10.1007/s00281-023-00992-6

**Published:** 2023-05-03

**Authors:** Jennifer E. Goertz, Lidia Garcia-Bonilla, Costantino Iadecola, Josef Anrather

**Affiliations:** Feil Family Brain and Mind Research Institute, Weill Cornell Medicine, 407 East 61St Street; RR-405, New York, NY 10065 USA

**Keywords:** Border-associated immune cells, Brain, Meninges, Choroid plexus, Calvaria, Skull bone marrow, Cirumventricular organs, Stroke, Hypertension, Neuroinflammation

## Abstract

Recent evidence implicates cranial border immune compartments in the meninges, choroid plexus, circumventricular organs, and skull bone marrow in several neuroinflammatory and neoplastic diseases. Their pathogenic importance has also been described for cardiovascular diseases such as hypertension and stroke. In this review, we will examine the cellular composition of these cranial border immune niches, the potential pathways through which they might interact, and the evidence linking them to cardiovascular disease.

## Introduction

The skull and brain feature several distinct border-associated immune compartments that contribute to immune homeostasis and cardiovascular disease. These compartments consist of the cranial bone marrow, dural meninges, leptomeninges, parenchymal perivascular space (Virchow-Robin space), circumventricular organs, choroid plexus, and brain ventricles. They can be considered immune compartments because they are separated from each other by structural and functional barriers that limit the free diffusion of inflammatory mediators and the trafficking of immune cells between each other and the brain parenchyma. Generally, these barriers are established by cell monolayers that are connected via tight junctions (TJs) to limit free diffusion of molecules and cell movement forming the blood–brain (endothelial TJ), CSF-brain (glia limitans/ependymal TJ), blood-CSF (choroid plexus epithelium TJ), dura-CSF (arachnoid TJ), and circumventricular organs (CVO)-CSF (tanycyte TJ) barriers. The structural organization of these barriers has been recently covered by several excellent reviews [1–4], and we will only discuss them as they pertain to immune functions.

## Border-associated immune compartments in brain and skull

### The skull bone marrow

Observations in humans indicate that the diploic space harbors hematopoietic bone marrow in children and young adults but gradually transforms to non-hemopoietic yellow bone marrow during adulthood. Based on postmortem anatomical exanimation and ^59^Fe uptake studies with radiologic evaluation, it has been estimated that 25% of the body’s total hemopoietic active bone marrow is localized in the skull of newborns and progressively declines to 7.6% at the age of 40 years [5]. In mice, the skull bone marrow is primarily confined to regions close to bone sutures and the base of the skull [6, 7]. scRNA-seq studies suggested that the bone marrow of the calvaria contained developing and adult immune cells in a similar composition to the tibial niche and that the immune cells showed comparable transcriptomes [7]. Despite these similarities in cellular composition, some differences in the transcriptomes of immune cells of the calvaria and the tibia were observed. Gene ontology analysis showed downregulation of proliferation genes in hematopoietic stem cells, genes associated with reactive oxygen species production in monocytes and macrophages, and genes related to myeloid cell differentiation in neutrophils when comparing the respective immune cell populations in the skull bone marrow to their counterparts in the tibia [7].

### The dura mater

The dura is one of the most diverse intracranial immune compartments. The immune cells can be divided into resident and transitory, although this distinction is not absolute, and it may depend on immune cell type and disease context.

#### Yolk sac-derived immune cells

Dural border-associated macrophages (BAMs) are tissue-resident macrophages located in close proximity of dural blood vessel [8] and are the most abundant cell type found in the dura [9, 10]. Although they share ontogeny with leptomeningeal and parenchymal macrophages [8], they differ in transcriptomic signatures showing higher MHC class II complex expression than leptomeningeal and parenchymal BAM [10]. MHC class II expression increases with age in mice possibly due to ongoing replacement of dural BAM by bone marrow-derived cells over the lifespan or due to changes in local environmental signals [10].

#### Bone marrow-derived immune cells

Multiple subsets of dendritic cells (DCs) have been detected in the dura [7, 10, 11]. Although conventional DC2 make up the majority of the dendritic cell population in the dura [10, 12], a smaller population of conventional DC1, migratory DC, and plasmacytoid DC are also present [10, 11]. In a study examining the origins of dural DC, adoptive transfer and parabiosis experiments indicated that pre-DCs from the periphery migrate to the meninges where they further differentiate into mature DC [13]. The half-life of dural DC was estimated to be 5–7 days. The results are in contrast with a more recent parabiont study that reported limited recruitment of dural DC from the periphery and implicated that the replenishment occurred primarily from the skull bone marrow and not the circulation [14].

Ly6c^hi^ monocytes and neutrophils are additional myeloid cell populations found in the dura under homeostatic conditions [7, 10, 11]. Dural monocytes and neutrophils seem to be preferentially recruited from the skull bone marrow via transosseous channels and do not maintain the dural pool by local proliferation [14]. The fate of these cells is unclear as they do not use lymphatic conduits to exit the cranium [14].

T cells, including conventional αβ T cells and γδ T cells, NK cells, and innate lymphoid cell type 2 (ILC2) have been described as constituents of the dural immune compartment [10, 11, 15, 16]. Conventional T cells are preferentially localized near dural sinuses, a special organization thought to be important for interaction with antigen presenting cells and lymphatic trafficking. Attraction and retention of T cells in the dural space might be aided by the expression of VCAM1, ICAM1, and Cxcl12 on stromal and endothelial cells [11]. In contrast to dural monocytes, neutrophils, and DC, parabiosis experiments indicate that dural T cells are recruited preferentially from the peripheral blood and not from the skull bone marrow [14]. γδT cells are found in the dura and have been suggested to originate from peripheral sources including the intestine [16]. Similar to T cells, ILC2 are found preferentially around the dural sinus and when compared to lung ILC2, dural ILC2 exhibit lower expression of genes related to inflammation, signal transduction, and metabolism, indicating a more quiescent state [15]. Based on the observation that ILC2 are a long lived tissue-resident immune cell population in other organs, it could be extrapolated that they have a slow turn over with a half-life of more than 4 months in some tissues [17]. This notion is supported by EdU pulse-chase experiments that detected no appreciable drop in ILC2 4 weeks after EdU administration [18].

There is also a prominent population of B cells in the dural meninges that has been found in rodents and primates [19, 20]. Investigations employing immunohistochemistry identified extravascular B cells in the dura, in addition to B cells within the vasculature and lymphatic vessels [21]. These cells tend to preferentially home near the venous sinuses [19, 21]. The studies further investigated the stage of development of these B cells and found dural progenitor populations that were similar in constitution to the ones present in the skull bone marrow. Furthermore, parabiosis experiments indicate that the majority of dural B cell progenitors originate from the skull bone marrow with only partial recruitment from other niches [19] and take advantage of the skull bone marrow-dura channels for transit [21]. Using B cells expressing Mog-specific immunoglobulin chains, it was shown that dural B cells but not B cells of the skull bone marrow or femur were reduced in these mice when compared to wild-type mice, suggesting that the dural niche is important for controlling CNS autoreactive B cells [20]. However, the presence of peripherally educated B cells in the dura has also been reported [22]. Using B cell receptor sequencing, it was found that dural plasma cells predominantly secreted IgA and that their B cell receptor repertoire was similar to that seen in plasma cells of the small intestine, suggesting an intestinal origin of these cells [22].

Besides dural BAM, the other resident meningeal immune cell population predominantly located in the dura of humans and rodents are mast cells [23, 24]. They develop from bone marrow-derived precursors that populate the CNS during development but are found mainly in the ependymal linings of the ventricles and the meninges in the adult where they are recruited from blood precursors [25]. Although the life span of dural mast cells has not been investigated, it is assumed that they can live for weeks to several month based on observations in other tissues [26, 27].

### Subdural meninges and perivascular space

While structurally and anatomically distinct, we will discuss these two compartments largely as a single unit because they are in close communication with each other and, to date, there are no pharmacologic or genetic tools to distinguish immune cell ontogeny and function within these compartments. As in the dura, the major immune cell type are resident macrophages, which are ontogenetically identical to dural macrophages and can be divided into subdural (leptomeningeal) BAM and perivascular macrophages (PVM) by their anatomical location but not by their transcriptomic profiles [28]. Subdural BAM share a similar transcriptomic profile and surface marker expression with MHCII^low^ dural BAM but some differences were also observed including higher *Lyve1* and *P2rx7* expression in subdural BAM [10]. While all PVM express CD206, LYVE-1 expression might be region specific. Immunohistochemical analysis showed that perivascular LYVE-1 immunoreactivity was highest around vessels of the hippocampus, although the study did not address whether this is due to increased expression or increased vascular PVM coverage in this brain region [29]. In the mouse, the parenchymal perivascular space along penetrating arterioles and venules is seeded with PVM after birth, most likely in conjunction with the development and expansion of the perivascular space [28]. These cells have a slow turnover rate, approximating that of microglia [10]. PVM play important roles in regulating paravascular CSF flow, vascular extracellular matrix deposition, and phagocytosis of potentially detrimental proteins [30].

The presence of other immune cells in the subdural meninges under homeostasis is less clear. This is based on the fact that collecting leptomeninges in mice without contamination from adjacent structures (brain, dura) is technically challenging. Moreover, the subarachnoid space under the parietal bone, the most commonly used location for in vivo imaging, extends only 30–80 µm in a healthy mouse which renders it difficult to attribute cells under observation to a specific compartment [31]. Recent developments in whole body tissue clearing combined with light sheet fluorescent microscopy (vDISCO [32]) have the potential to overcome some of these limitations and similar techniques have been applied to study the vascular structure and bone development of the calvarium [33] and the meningeal lymphatic vasculature [34].

### Choroid plexus (ChP)

Like microglia and CNS border-associated macrophages, ChP macrophages in stroma and at the apical side of the ChP epithelium (epiplexus macrophages) are yolk-sack derived and seed the brain early during development [35]. Compared to other brain myeloid cells, stromal ChP macrophages are short-lived (weeks in the adult mouse) and are at least partially replaced by blood monocyte-derived macrophages [36, 37]. In contrast, similar to microglia and PVM, epiplexus macrophages are self-regenerating without contribution of the bone marrow niche [10]. Developmentally the ChP niche is seeded by CD206^high^ (*Mrc1*) macrophages. Over the life span of the mouse CD206^high^ stromal macrophages give way to CD206^low^MHCII^high^ macrophages, although it is unclear if this occurs due to a transcriptional switch in resident cells or if this reflects ongoing replacement by blood monocyte-derived cells [10, 35]. Other cells found in the ChP stroma are lymphocytes, NK cells, monocytes, neutrophils, and DC, although at significantly lower numbers than resident macrophages [10, 35].

### Circumventricular organs

The CVO-brain and CVO-CSF barrier is formed by tanycytes, specialized glial cells that line the third and parts of the fourth ventricle, and specialized astrocytes [38–40]. Given their lack of a structural BBB, macromolecules can freely diffuse into the parenchyma of CVO. Vessel-associated myeloid cells, including possibly PVM, are found in higher numbers in CVO than in other brain regions [41] and are able to sequester blood derived macromolecules [42]. In addition to resident BAM, peripheral immune cells are found in CVO under homeostatic conditions. Reconstitution of Rag2^–/–^ lymphopenic mice with GFP^+^ T cells let to robust seeding of the ChP, median eminence (ME), vascular organ of the lamina terminalis (OVLT), and the subfornical organ (SFO) but not the brain parenchyma [43]. T and NK cells have been detected in the ME of mice under homeostatic conditions by scRNA-seq [44]. Similarly, pathology specimens of humans without CNS disorder showed elevated numbers of CD8^+^ T cells in ventral medulla oblongata and area postrema [45].

## CSF and the cranial vascular network as barriers and facilitators of intercompartmental communications

The brain is surrounded by cerebrospinal fluid (CSF) which is produced at high rates (~ 500 ml per day in humans and ~ 140 µl in the mouse) [46] primarily by the ChP epithelium. CSF is drained from the subarachnoid space through the intracranial and spinal venous system but is also collected by intracranial lymphatic vessels or drains along perineural sheets of cranial nerves to reach extracranial and nasal tissues [47, 48]. Under homeostatic conditions, the CSF contains few innate immune cells, while effector and memory T cells constitute the largest population [49]. The migration pattern of these cells has not been fully elucidated. Extravasation of effector T cells from pial vessels and ChP and transarachnoid migration from the dural extracellular space have been observed, and the relative contribution of these pathways might be disease dependent [2, 50]. Under homeostatic conditions, CSF T cells are transitory [49] and egress from the CSF through dural lymphatic vessels to reach deep cervical lymph nodes and through transcribrosal lymphatics to reach the superficial cervical lymph node (LN) [51]. Owing to endothelial fenestrations and lack of TJ, the blood vessel of the dura mater, choroid plexus, and CVO are leaky, resulting in free exchange of macromolecules between the blood and the surrounding tissue. This increased permeability also facilitates the egress of peripheral immune cells into surrounding structures.

Anatomically, the cranial vasculature can be divided into four major districts: pericranial, diploic, meningeal, and cerebral. The majority of the cerebral venous outflow drains into the dural sinuses from where it exits the cranium through the internal jugular vein in humans, while the external jugular vein is the major outflow path in rodents [52, 53]. Therefore, cerebral veins need to transverse the subarachnoid space and penetrate the dura to connect to the draining venous system.

In primates, as in rodents, the diploe between the outer and inner tables of cranial bones is vascularized by an intricate network of thin-walled diploic veins that show formation of lacunar structures. These veins drain mainly to the dural sinuses but connections to the extracranial veins of the scalp by emissary veins are also present [54]. The diploic veins in mice increase both in covered territory and complexity with age [55]. Since the diploic veins, similar to other intracranial veins, are devoid of valves, the blood in these vessels can theoretically flow in both directions, although in a mouse study the flow was directional from the dural layer to the diploic veins [55].

Whereas pia and arachnoid do not have a dedicated blood supply, the dural arterial network is largely provided by branches of the middle meningeal artery, which in rodents originates from the pterygopalatine or stapedial arteries, both of which are branches of the internal or common carotid arteries. By contrast, in primates, the middle meningeal artery arises from the maxillary artery, a branch of the external carotid artery [56]. This could have implications when modeling human disease in rodents.

### Communications between the subarachnoid and dural space

Several studies using intracerebroventricular, lumbar, or cisterna magna injections of tracers have shown functional connections between the subarachnoid space, the dura, and the diploe in rodents and humans [7, 11, 57–59]. However, the anatomical structures underlying these communications are less well defined and therefore debated. Arachnoid granulations are protrusions of villi-like structures of the arachnoid membrane into the dural sinuses and have long been considered as one of the main drainage systems for CSF [60, 61]. They connect with the endothelial layer of the dural sinus that at that point is devoid of the fibrous collagen-rich layers that are characteristic for the dura. Arachnoid granulations can also protrude into parasagittal venous lacunae that receive inputs from diploic and emissary veins [62]. More recently, arachnoid granulations protruding into the diploic space of the calvaria have been described in humans and have been implicated in CSF absorption through contact with diploic veins [63], and a potential role of this efflux pathway for immune signaling in humans has been proposed [58]. Interestingly, arachnoid granulations show marked diversity in location, structure, appearance, and cellular composition and the presence of CD4^+^ T cells and CD11c^+^ myeloid cells in the stroma of some types of arachnoid granulations suggest possible immune function of these structures [64]. Although “bona fide” arachnoid granulations are thought to be absent in rodents, meningeal invaginations into dural venous sinuses that could serve as CSF outflow pathways have been described in mice [65]. CSF could also be transported along arachnoid sheets that protrude into the dural space along penetrating cerebral veins which connect the subdural veins with the dural sinuses [66]. Perivascular routes of tracer dissemination to the dura and diploe after cerebroventricular injections have been described in mice [59].

The brain itself is covered by a continuous layer of astrocyte cell bodies in rodents or by cytoplasmic processes of marginal astrocytes in primates known as the glia limitans, whereas processes of Bergman glia form the glia limitans of the cerebellum [67]. The astrocytes are in close contact with the basal lamina, which is in intimate contact with the pia. This structure forms a tight barrier to the subarachnoid space and limits the exchange of macromolecules and cells between the brain parenchyma and the CSF. The glia limitans extends along penetrating blood vessels, and together with basement membranes and endothelial TJ forms the BBB that renders the blood vessels of the cerebral vasculature impermeable to macromolecules and circulating cells. However, the glia limitans superficialis is largely absent at the olfactory bulbs [67], and together with a discontinuous arachnoid membrane along the cribriform plate [68], the rostral cranial compartment could constitute a site of exchange for macromolecules and possibly immune cells between the brain parenchyma, CSF, and the dura.

### Communication between dural and diploic compartments

There are an estimated 1000 channels in the calvaria of the adult mouse connecting the dura to the bone marrow of the calvaria. They are approximately 80–100 µm in length and have a diameter about 20 µm [69]. Using endothelial and vascular smooth muscle markers, it was established that these channels contain blood vessels [70]. Furthermore, venous tracing studies found that small transosseous vessels connected the dural venous with the diploic venous system possibly by way of these channels [55]. The space surrounding these vessels can carry CSF as shown by the observation that intracisternally injected tracer can be detected in the perivascular space within the bone marrow. Two thirds of imaged channels exhibited perivascular tracer signal, but it is unclear whether CSF can flow through all channels [59]. This paravascular space is also utilized by skull bone marrow myeloid cells to seed the dural space [69].

Collectively, these studies provide support for the existence of anatomically and functionally distinct border-associated immune compartments in the cranium. CVO, ChP, dura mater, and skull bone marrow, while preserving their immunologic identity, are in close communication with circulating immune cells consistent with their lack of a functional BBB. In contrast, the brain ventricles, leptomeninges and parenchymal perivascular spaces are largely shielded from the periphery under homeostatic conditions, and their resident immune cell populations are either long-lived or replenished by autonomous cell replacement similar to the one observed in microglia [36].

## The cranial border-associated immune compartments in aging and disease

### Age-associated changes in cranial immune compartments

Aging is the major non-modifiable risk factor for cardiovascular and cerebrovascular diseases. Age-related changes have been reported across all cranial border-associated immune compartments. B cell progenitors may be less frequent in the aged dural meninges [20]; however, there appears to be an overall increase in total B cells in the dura [21]. This increase is associated with an increased clonal overlap with blood-based B cells and plasma cells, indicating there may be replacement from the periphery over time [21]. In the dura, both total CD4^+^ and CD8^+^ T cells also increase in aging, and their spatial organization changes such that they are primarily localized away from venous sinuses [11]. No age-associated changes have been identified in dendritic cell populations [11]. There is an increase in neutrophils and macrophages in brains of aged mice [71]. These macrophages seem to be involved in an inflammatory feedforward cycle with brain-resident microglia [71]. In monkeys, there is an age-related increase in CD3^+^ T cells in the ChP, meninges, and perivascular spaces of parenchymal vessels [72]. Perivascular T cell infiltrates were primarily located in the white matter, but an increase of parenchymal T cells was also observed. This did not seem to be due to breakdown of the BBB, but it was associated with increased density of inflammatory microglia and cognitive decline [72]. Similar findings were also reported in aged mice where CD3^+^ T cells were predominantly located in perivascular spaces of white matter tracts [73]. Aging could shift immune cell composition of the skull bone marrow in mice resulting in increased cytotoxic T cells and CD11b^high^ B cells [74]. An expansion of dura and ChP resident ILC2 has also been observed in aging and linked to better cognitive performance [18].

### Effects of stroke on cranial immune compartments

#### Skull bone marrow

The cellular composition of the skull bone marrow changes after experimental cerebral ischemia to a higher degree than in other bone marrow compartments. Six hours after focal ischemia produced by transient occlusion of the middle cerebral artery (MCA), the skull marrow contained significantly fewer neutrophils and monocytes than the tibia, possibly reflecting egress of myeloid cells from the skull to the meninges [69].

#### Meninges

**Fig. 1:**
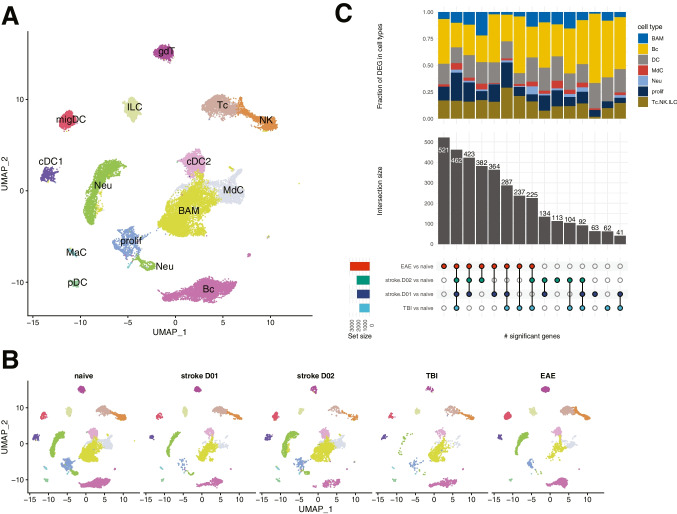
Similarities and differences in the immune response of dural immune cells across different disease models. Single-cell RNA-seq data were compiled from GEO (https://www.ncbi.nlm.nih.gov/geo/) submissions GSE128854 [10], GSE144175 [11], GSE178085 [113], GSE189432 [77], GSE206940 [114], and an unpublished in-house dataset from mice 2 days after transient MCA occlusion. Only samples with libraries from naïve mice, mice undergoing EAE (experimental autoimmune encephalitis), TBI (mild traumatic brain injury), or stroke (transient MCA occlusion) were included. The data was further filtered to select libraries generated from immune cells of the dural meninges from 7 to 16 weeks old mice. **A** UMAP of major immune cell types; border-associated macrophages (BAM), B cells (Bc), conventional DC type 1 (cDC1), conventional DC type 2 (cDC2), γδT cells (gdT), innate lymphoid cells (ILC), mast cells (MaC), monocyte-derived cells (MdC), migratory dendritic cells (migDC), neutrophils (Neu), natural killer cells (NK), plasmacytoid dendritic cells (pDC), proliferating cells (prolif), T cells (Tc). **B** UMAP split by condition; **C)** Upset plot showing overlapping and disease-specific differentially expressed genes (DEG) in response to autoimmunity, mild traumatic brain injury, or stroke

There are also changes to the structure of the meninges after stroke, but the effect could be model dependent. Photothrombotic stroke results in meningeal lymphangiogenesis, a change not observed in transient focal ischemia [75]. This effect appears to be conferred by VEGFR-3, as heterozygous mutant mice had significantly larger infarct volumes after transient ischemia, whereas no difference was observed in photothrombotic stroke [75]. Whether the deleterious effects of VEGFR-3 haploinsufficiency is due to defective lymphatic outflow from the cranium or due to altered systemic lymphangiogenesis and its effect on the peripheral immune system has yet to be determined. Interestingly, acute inhibition of VEGFR-3 signaling by intranasal delivery of a receptor antagonist or lymphadenectomy of superficial LN in rats decreased brain injury after transient ischemia [76]. Flow cytometry analysis revealed that meningeal B and T cell populations were unchanged at both 24 and 72 h after transient MCA occlusion when compared to mice undergoing sham surgery [77]. In the pia, granulocytes were increased at 24 h and subsequently returned to normal levels by 72 h. There was also a trend that dendritic cells were decreased at both time points, reaching significance in the dura. Dural NK cells were decreased at 24 h and mostly recovered at 72 h, a pattern that was also found in the pia, though without significance. In a mouse model of subarachnoid hemorrhage (SAH), parenchymal perivascular spaces were enlarged and T cells were increased in the meninges 7 days after injury [78]. Gene expression of immune cells in dural meninges is altered by injury and is dependent on the disease model. We reanalyzed publicly available scRNA-seq data from naïve mice and mice after ischemic stroke, mild traumatic brain injury, or experimental autoimmune encephalomyelitis (EAE). This analysis showed while the overall qualitative composition of immune cell clusters found in the dura was not changed, there were overlapping and disease-specific changes in the transcriptome (Fig. 1). EAE showed the highest number of uniquely differential regulated genes when compared to naïve animals whereas the majority of differential regulated genes in the other disease models were shared across conditions and only few were disease specific (Fig. 1C). This may reflect the different pathoetiology of EAE which is mainly characterized by an adaptive immune response while stroke and traumatic brain injury are, at least in the acute phase, driven by innate immunity.

Suggestive of a local expansion of BAM after ischemic brain injury, a histological investigation in rats found an increase in cells expressing the BAM marker CD163^+^, in the leptomeninges 3 days after transient MCA occlusion [79]. These cells were also positive for the proliferation marker Ki67 [79]. Another study in rats also examining CD163^+^ cells suggested this increase began not earlier than 24 h after ischemia [80]. Similarly, using CD206 as a BAM marker, it was found that BAM increased in meninges and the perivascular space along penetrating vessels from day 1 to 3 after transient MCA occlusion in rats; however the study suggested migration from the meninges as the main mechanism for this increase [81]. Interestingly, a study of six human cases with a history of MCA infarction found increased MHC class II positive PVM in the spinal cord weeks to several years after the ischemic event. The PVM were also rich in lipid droplets indicating that these macrophages might be involved in the pyramidal tract degeneration observed after ischemic brain injury [82]. Whether these cells contribute to Wallerian degeneration or constitute a reactive response remains to be established, but the study suggests that after stroke, PVM could play a role in neurodegeneration or repair far away from the site of primary injury.

#### Choroid plexus

The ChP has also been identified as a target organ after ischemic and hemorrhagic stroke [83]. Focal ischemia in rats resulted in a proliferative response as early as 2 h after injury, although immune cells were not investigated in this study [84]. In addition, intraluminal MCA occlusion models might decrease blood flow in the lateral ventricle ChP, produce ChP edema [85] and induce cell death in the ipsilateral ChP [86]. Using a model of distal MCA occlusion in mice, it was shown that expression of *Vcam1*, *Cx3cl1*, *Madcam1*, and *Nt5e* (CD73) was increased after stroke [87], indicating that activation of the ChP can also occur in stroke models that do not produce a lesion close to the ChP such as the intraluminal filament model. Using flow cytometry it was shown that monocyte-derived cells increased in the ChP 24 and 72 h after stroke whereas NK cells were reduced [77]. In a model of neonatal stroke, monocytes and neutrophils were recruited to the ChP as early as 3 h after injury and leukocyte infiltration was partially impaired in mice lacking the scavenger receptor CD36 [88].

### Involvement of cranial border immune compartments in stroke

#### Border-associated macrophages

The role of BAM in different stroke models has been mainly addressed by depleting BAM with intracerebroventricularly delivered clodronate liposomes, which are selectively taken up by BAM resulting in apoptotic cell death [89, 90]. A major limitation of this approach is that BAM are depleted in all meningeal and ChP compartments, making it impossible to attribute observed effects to a specific immune niche. Moreover, effects of clodronate liposomes on other immune cells of the cranium, including dural monocytes/neutrophils and those of the skull bone marrow, cannot be excluded, especially under disease conditions when the phagocytic activity of myeloid cells might be increased.

When BAM were depleted 4 days before ischemia/reperfusion in rats, there was no difference in infarct volume, and there were no significant differences in parenchymal infiltration of T cells, NK cells, or monocytes while recruitment of neutrophils to the ischemic cortex, and disruption of the BBB was diminished [80]. In a study of SAH, clodronate treatment 3 h after SAH induction resulted in reduced sensorimotor deficits, but had no effect when BAM were depleted before SAH induction [91], possibly indicating a protective effect of BAM during the hyper-acute phase of injury or effects of clodronate on peripheral monocytes infiltrating the site of injury. Neutrophils primarily seed the dura after SAH while monocytes and T cells were found in the parenchyma. The infiltration of neutrophils and cognitive impairment after SAH was dependent on their expression of myeloperoxidase, whereas deletion of the essential p47-phox subunit of the NADPH oxidase complex, a major source of radicals, or deletion of neutrophil elastase had no effect [92]. Adding additional complexity, the roles of BAM in stroke might be context dependent. In a model of chronic alcohol exposure in mice, which induces microglia and endothelial inflammatory priming, it was found that depletion of BAM prevented the aggravating effect of alcohol on ischemic brain injury while it had no effects in stroke mice not exposed to alcohol [93].

#### Dural mast cells

Because mast cells contain granules with vasoactive molecules and proteases, they have been implicated in BBB disruption and in promoting neutrophil extravasation in the course of cerebral ischemia [24, 94, 95]. Using c-kit mutant mice that show mast cells deficiency, it was shown that depletion of mast cells resulted in decreased levels of neuroinflammation, brain edema, neutrophil infiltration, and infarct size following stroke [95, 96]. Repopulating meningeal mast cells by transfer of in vitro generated bone marrow-derived mast cells reverted the protective phenotype in c-kit mutant mice, and this was partially dependent upon the ability of mast cells to produce IL-6 [95]. Similarly, preventing mast cell degranulation by intracerebroventricular delivery of cromoglycate reduced brain edema and neutrophil infiltration both in ischemic and hemorrhagic stroke models [94, 96, 97].

#### T cells

Dural T cells are continuously recruited from the periphery [14] including the small intestine [16, 98]. Whether the local dural environment affects their phenotype has not been investigated, but there seems to be retention of the activation state imprinted in the periphery. For example, while the number of total dural γδT was not changed in mice with altered microbiota after stroke, they produced more IL-17, which was mirrored by increased IL-17 production in γδT cells in the small intestine. While γδT cells were not found in the brain parenchyma during the acute phase of ischemic brain injury, the study suggested that IL-17 produced in the dura could affect stroke outcome by enhancing parenchymal neutrophil recruitment [16].

#### The roles of leptomeninges and ChP in the immune cell recruitment after stroke

Several studies have addressed the role of leptomeninges as a source of blood-borne immune cells after stroke [77, 99–103]. Consistent with a meningeal origin, neutrophils are found on the abluminal site of leptomeningeal vessel within hours after stroke in permanent and transient ischemia models in rodents. A strong association of neutrophils with leptomeningeal vessel has also been observed in tissue samples from human stroke victims [101]. Whether neutrophils that extravasated in the subarachnoid and perivascular space go on to infiltrate the ischemic territory remains to be established, but the fact that accumulation in the leptomeninges precedes the appearance of neutrophils in the brain parenchyma supports such a scenario [99–101]. The leptomeninges showed accumulation of macrophages and neutrophils within 1 day after permanent distal MCA occlusion in spontaneously hypertensive rats, suggestive of a meningeal role in the immune cell infiltration after stroke [102]. In support of these findings, a study in mice specifically addressing the distribution of neutrophils between different cranial immune compartments after transient focal ischemia found that neutrophils were increased in the leptomeninges and perivascular spaces early after ischemia preceding their accumulation in the brain parenchyma [103]. In contrast to leptomeninges, dural neutrophils did not change after stroke [77].

The ChP is also involved in the recruitment of monocytes and T cells after stroke. Using cell tracking in mice, monocyte-derived cells were found in the ChP and CSF one day after focal ischemia [87]. The number of cells in the CSF was decreased at day 3 after stroke while the number of labeled monocyte-derived cells in the ischemic territory was increased at this time point, suggesting a ChP-CSF-brain route for the recruitment of these cells [87]. Photothrombotic lesioning of ChP reduced T cell infiltration into the ipsilateral cortex after permanent distal MCA occlusion in mice [86]. Interestingly, blocking of CSF flow by Matrigel injection into the ipsilateral ventricle did not significantly alter T cell counts in the ischemic hemisphere, indicating that migration via the ventricular space might not constitute the main route for T cell recruitment to the ischemic territory. It was proposed that direct parenchymal infiltration from the ChP into adjacent brain structures and dissemination along callosal white matter tracts allows T cells to reach the periinfarct regions [86].

The evidence presented above underlines the complex roles of border-associated immune compartments in ischemic brain injury, and while immune cells of those compartments seem not to be involved in promoting tissue injury, they might play an important role in orchestrating the inflammatory response of brain resident and infiltrating immune cells.

### Cerebral amyloid angiopathy (CAA)

Due to their perivascular localization and phagocytic capacity, PVM are particularly suited to control the flux and participate in the clearance of potentially harmful molecules such as Aβ aggregates. BAM depletion in an early-onset mouse model of Alzheimer’s disease (AD) with parenchymal Aβ plaques and CAA like pathology (TgCRND8 mice), increased perivascular Aβ deposits one month after intracerebroventricular clodronate administration suggest that BAM are involved in vascular Aβ clearance [104]. In contrast, BAM might contribute to neurovascular dysfunction and cognitive impairment in a late-onset mouse AD model (Tg2576). Depleting BAM at 3 months of age, when there was neurovascular impairment but no Aβ plaques, resulted in reversal of neurovascular dysfunction. The deleterious effects of BAM in this model were linked to increased oxidative stress induced by Aβ signaling through the scavenger receptor CD36 resulting in NADPH oxidase activation, reactive oxygen species production, and cerebrovascular dysfunction [105].

Taken together, these studies show that the contribution of BAM to cerebrovascular pathologies found in AD might be disease stage dependent, being a driver of cerebrovascular dysfunction during the prodromal stages while contributing to Aβ clearance once intramural Aβ accumulation has occurred.

### Systemic cardiovascular diseases

Endothelial dysfunction has a major impact on the bone marrow hematopoietic niche, as demonstrated in mouse models of hypertension, atherosclerosis, and myocardial infarction [106]. In the skull bone marrow of mice with hypertension, the study found increased angiogenesis, perivascular collagen deposition, and integrin expression resulting in augmented leakiness and endothelial dysfunction. These changes in the vascular niche were associated with increased hematopoietic progenitor proliferation and higher numbers of circulating innate immune cells [106]. Hypertension might also induce shifts in intracranial immune cell distribution. For example, middle-aged spontaneous hypertensive rats (SHR) showed accumulation of intravascular NK and T cells in the microvasculature of the brain parenchyma when compared to normotensive rats in which most NK and T cells are found in the ChP and meninges. These changes were correlated with increased endothelial VCAM1 and decreased P-selectin expression [107]. SH rats also showed increased numbers of PVM in the brain parenchyma [108]. Elimination of BAM by intracerebroventricular injection of clodronate liposomes diminished neuronal activity in the periventricular nucleus of the hypothalamus and the rostral ventrolateral medulla, two brain nuclei involved in blood pressure regulation, and decreased the blood pressure elevation observed in SH rats between 8 and 10 weeks of age without affecting peripheral inflammation as reflected by circulating Il-1β levels [108]. The study also found that the blood pressure and sympathetic tone increase after systemic IL-1β administration was attenuated in rats with intracranial BAM depletion, indicating that hypertension upon peripheral inflammation might depend on BAM to relay systemic signals to cardiovascular centers in the brain. Similarly, the sympathetic activation and blood pressure increase observed after intracarotid TNF injection was abrogated in normal rats treated with intracerebroventricularly delivered clodronate liposomes. It was suggested that this response was dependent on cyclooxygense-2 mediated PGE_2_ production in PVM of the paraventricular nucleus which increased neuronal activity leading to an elevated sympathetic tone [109].

This is in contrast with studies in spontaneously hypertensive mice (BPH/J2) and mice with slow pressor Ang II hypertension where clodronate BAM depletion had no effect on systemic blood pressure but ameliorated BBB dysfunction and improved cognitive performance [110, 111]. This effect was partially dependent on the presence of angiotensin type 1A receptors and NADPH oxidase activity in BAM.

Salt-induced hypertension results in neurovascular dysfunction and cognitive impairment [110]. A recent study identified dural IL-17-producing γδ T cells and PVM as major drivers of the dysfunction observed in this model. It was shown that intracranial IL-17 production by dural T cells triggered increased superoxide production in PVM through IL-17RA signaling. Because superoxide is highly reactive with nitric oxide, a major endothelium-derived vasodilator, this resulted in decreased nitric oxide bioavailability leading to impaired neurovascular coupling and cognitive performance without affecting systemic arterial blood pressure [112].

## Conclusions and future directions

**Fig. 2:**
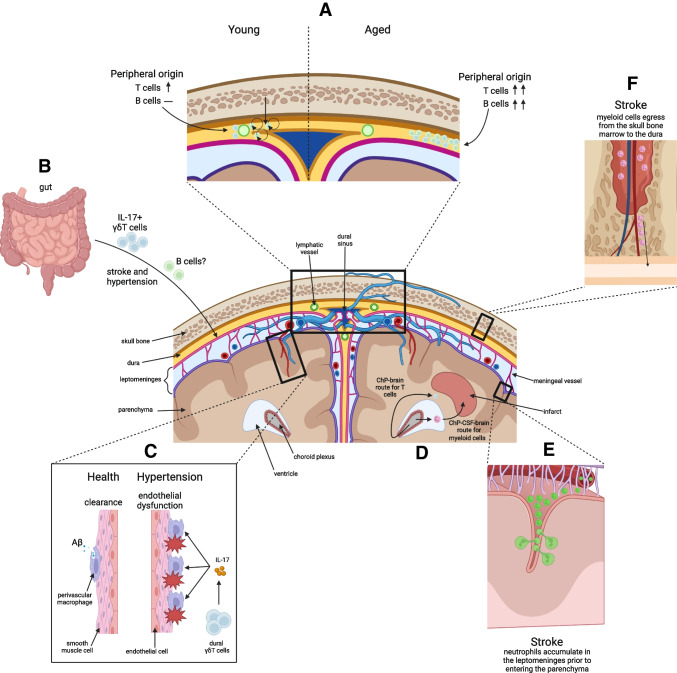
Effects of age and cerebrovascular disease on border immune compartments of the cranium. **A** In young mice, self-proliferating B cells originating from the skull bone marrow and T cells originating from the periphery organize around the lymphatic vessels and sinuses in the dura. In aged mice, these cells originate only from the periphery and are increased in abundance, organizing away from dural sinuses. **B** Some of the dural B cells might be entrained in the gut and IL-17 producing γδT cells migrate from the gut to the meninges following stroke or hypertension. **C** Perivascular macrophages line penetrating vessels of the brain. In health, these macrophages are beneficial in clearing debris and facilitating paravascular fluid movement. In hypertension, they can promote endothelial dysfunction through ROS production. **D** Myeloid cells follow a ChP-CSF-brain route to infiltrate the infarct, whereas T cells enter the brain parenchyma adjacent to the choroid plexus to reach the peri-infarct region. **E** Neutrophils accumulate in the leptomeninges following stroke before they extravasate into the parenchyma. **F** After stroke, myeloid cells egress from the skull bone marrow into the dura

The border-associated immune compartments of the brain, its coverings, and the skull are embedded in structures that allow molecular and cellular communication between themselves and the systemic circulation (Fig. 2). Although their roles in the pathophysiology of cardiovascular diseases are increasingly appreciated, several central questions remain to be addressed. Assuming that the localization to intracranial immune compartments is not stochastic, what are the signals for immune cell recruitment and retention to CVO, ChP, and dura? What is the impact of ongoing replacement of resident immune cells by blood-derived cells during aging on the function of these compartments and their roles in cardiovascular disease? How are signals from these border-associated immune compartments transmitted to the brain? What is the effect of cardiovascular disease and co-morbidities on the composition, function, and reactivity of these immune niches? Answering some of these questions will provide new insights into the function of border-associated immune compartments of the cranium and provide the conceptual framework for the development of therapeutic approaches to target these immune niches.

## References

[1] Alves de Lima K, Rustenhoven J, Kipnis J (2020). Meningeal immunity and its function in maintenance of the central nervous system in health and disease. Ann Rev Immunol.

[2] Mastorakos P, McGavern D (2019). The anatomy and immunology of vasculature in the central nervous system. Sci Immunol.

[3] Engelhardt B, Vajkoczy P, Weller RO (2017). The movers and shapers in immune privilege of the CNS. Nat Immunol.

[4] Coles JA (2017). Where are we? The anatomy of the murine cortical meninges revisited for intravital imaging, immunology, and clearance of waste from the brain. Prog Neurobiol.

[5] Cristy M (1981). Active bone-marrow distribution as a function of age in humans. Phys Med Biol.

[6] Kosaras B (2009). Sensory innervation of the calvarial bones of the mouse. J Comp Neurol.

[7] Mazzitelli JA (2022). Cerebrospinal fluid regulates skull bone marrow niches via direct access through dural channels. Nat Neurosci.

[8] Sato T (2021). Morphology, localization, and postnatal development of dural macrophages. Cell Tissue Res.

[9] Mrdjen D (2018). High-dimensional single-cell mapping of central nervous system immune cells reveals distinct myeloid subsets in health, aging, and disease. Immunity.

[10] Van Hove H (2019). A single-cell atlas of mouse brain macrophages reveals unique transcriptional identities shaped by ontogeny and tissue environment. Nat Neurosci.

[11] Rustenhoven J (2021). Functional characterization of the dural sinuses as a neuroimmune interface. Cell.

[12] Mundt S (2019). Conventional DCs sample and present myelin antigens in the healthy CNS and allow parenchymal T cell entry to initiate neuroinflammation. Sci Immunol.

[13] Anandasabapathy N (2011). Flt3L controls the development of radiosensitive dendritic cells in the meninges and choroid plexus of the steady-state mouse brain. J Exp Med.

[14] Cugurra A (2021). Skull and vertebral bone marrow are myeloid cell reservoirs for the meninges and CNS parenchyma. Science.

[15] Gadani SP (2016). Characterization of meningeal type 2 innate lymphocytes and their response to CNS injury. J Exp Med.

[16] Benakis C (2016). Commensal microbiota affects ischemic stroke outcome by regulating intestinal gammadelta T cells. Nat Med.

[17] Schneider C (2019). Tissue-resident group 2 innate lymphoid cells differentiate by layered ontogeny and in situ perinatal priming. Immunity.

[18] Fung ITH et al (2020) Activation of group 2 innate lymphoid cells alleviates aging-associated cognitive decline. J Exp Med 217(4):e20190915 10.1084/jem.20190915 PMC7144523 32022838

[19] Schafflick D (2021). Single-cell profiling of CNS border compartment leukocytes reveals that B cells and their progenitors reside in non-diseased meninges. Nat Neurosci.

[20] Wang Y (2021). Early developing B cells undergo negative selection by central nervous system-specific antigens in the meninges. Immunity.

[21] Brioschi S (2021). Heterogeneity of meningeal B cells reveals a lymphopoietic niche at the CNS borders. Science.

[22] Fitzpatrick Z (2020). Gut-educated IgA plasma cells defend the meningeal venous sinuses. Nature.

[23] Dimlich RV (1991). Linear arrays of homogeneous mast cells in the dura mater of the rat. J Neurocytol.

[24] Lindsberg PJ, Strbian D, Karjalainen-Lindsberg M-L (2010). Mast cells as early responders in the regulation of acute blood-brain barrier changes after cerebral ischemia and hemorrhage. J Cereb Blood Flow Metab.

[25] Silver R, Curley JP (2013). Mast cells on the mind: new insights and opportunities. Trends Neurosci.

[26] Padawer J (1974). Mast cells: extended lifespan and lack of granule turnover under normal in vivo conditions. Exp Mol Pathol.

[27] Kiernan JA (1979). Production and life span of cutaneous mast cells in young rats. J Anat.

[28] Masuda T (2022). Specification of CNS macrophage subsets occurs postnatally in defined niches. Nature.

[29] Karam M (2022). Heterogeneity and developmental dynamics of LYVE-1 perivascular macrophages distribution in the mouse brain. J Cereb Blood Flow Metab.

[30] Drieu A (2022). Parenchymal border macrophages regulate the flow dynamics of the cerebrospinal fluid. Nature.

[31] Marchetti L, Engelhardt B (2020). Immune cell trafficking across the blood-brain barrier in the absence and presence of neuroinflammation. Vasc Biol.

[32] Cai R (2019). Panoptic imaging of transparent mice reveals whole-body neuronal projections and skull–meninges connections. Nat Neurosci.

[33] Rindone AN (2021). Quantitative 3D imaging of the cranial microvascular environment at single-cell resolution. Nat Commun.

[34] Jacob L et al. (2022) 3D-imaging reveals conserved cerebrospinal fluid drainage via meningeal lymphatic vasculature in mice and humans. bioRxiv. 10.1101/2022.01.13.476230

[35] Dani N et al (2021) A cellular and spatial map of the choroid plexus across brain ventricles and ages. Cell 184(11):3056–3074.e21 10.1016/j.cell.2021.04.003 PMC8214809 33932339

[36] Goldmann T (2016). Origin, fate and dynamics of macrophages at central nervous system interfaces. Nat Immunol.

[37] Cui J, Xu H, Lehtinen MK (2021). Macrophages on the margin: choroid plexus immune responses. Trends Neurosci.

[38] Langlet F (2013). Tanycyte-like cells form a blood–cerebrospinal fluid barrier in the circumventricular organs of the mouse brain. J Comp Neurol.

[39] Morita S (2016). Heterogeneous vascular permeability and alternative diffusion barrier in sensory circumventricular organs of adult mouse brain. Cell Tissue Res.

[40] Garcia-Caceres C (2019). Role of astrocytes, microglia, and tanycytes in brain control of systemic metabolism. Nat Neurosci.

[41] Takagi S (2019). Microglia are continuously activated in the circumventricular organs of mouse brain. J Neuroimmunol.

[42] Willis CL, Garwood CJ, Ray DE (2007). A size selective vascular barrier in the rat area postrema formed by perivascular macrophages and the extracellular matrix. Neuroscience.

[43] Song C (2016). Expansion of brain T cells in homeostatic conditions in lymphopenic Rag2−/− mice. Brain Behav Immun.

[44] Chen ZH (2022). Single-cell transcriptomic profiling of the hypothalamic median eminence during aging. J Genet Genomics.

[45] Loeffler C (2011). Immune surveillance of the normal human CNS takes place in dependence of the locoregional blood-brain barrier configuration and is mainly performed by CD3(+)/CD8(+) lymphocytes. Neuropathology.

[46] Liu G (2020). Direct Measurement of Cerebrospinal Fluid Production in Mice. Cell Rep.

[47] Louveau A (2015). Structural and functional features of central nervous system lymphatics. Nature.

[48] Ma Q (2017). Outflow of cerebrospinal fluid is predominantly through lymphatic vessels and is reduced in aged mice. Nat Commun.

[49] Hickey WF, Hsu BL, Kimura H (1991). T-lymphocyte entry into the central nervous system. J Neurosci Res.

[50] Merlini A (2022). Distinct roles of the meningeal layers in CNS autoimmunity. Nat Neurosci.

[51] Louveau A (2018). CNS lymphatic drainage and neuroinflammation are regulated by meningeal lymphatic vasculature. Nat Neurosci.

[52] Patel N (2009). Venous Anatomy and Imaging of the First Centimeter. Seminars in Ultrasound, CT and MRI.

[53] Mancini M (2015). Head and neck veins of the mouse. A magnetic resonance, micro computed tomography and high frequency color Doppler ultrasound study. PLoS ONE.

[54] García-González U (2009). The diploic venous system: surgical anatomy and neurosurgical implications. Neurosurg Focus.

[55] Toriumi H (2011). Developmental and circulatory profile of the diploic veins. Microvasc Res.

[56] Mecheri B, Paris F, Lübbert H (2018). Histological investigations on the dura mater vascular system of mice. Acta Histochemica.

[57] Ringstad G, Eide PK (2020). Cerebrospinal fluid tracer efflux to parasagittal dura in humans. Nat Commun.

[58] Ringstad G, Eide PK (2022). Molecular trans-dural efflux to skull bone marrow in humans with CSF disorders. Brain.

[59] Pulous FE (2022). Cerebrospinal fluid can exit into the skull bone marrow and instruct cranial hematopoiesis in mice with bacterial meningitis. Nat Neurosci.

[60] Upton ML, Weller RO (1985). The morphology of cerebrospinal fluid drainage pathways in human arachnoid granulations. J Neurosurg.

[61] Kapoor KG (2008). Cerebrospinal fluid outflow: an evolving perspective. Brain Res Bull.

[62] Cure JK, Van Tassel P, Smith MT (1994). Normal and variant anatomy of the dural venous sinuses. Semin Ultrasound CT MR.

[63] Tsutsumi S (2014). Cranial arachnoid protrusions and contiguous diploic veins in CSF drainage. Am J Neuroradiol.

[64] Shah T (2023). Arachnoid granulations are lymphatic conduits that communicate with bone marrow and dura-arachnoid stroma. J Exp Med.

[65] Mollgard K (2023). A mesothelium divides the subarachnoid space into functional compartments. Science.

[66] Krisch B (1988). Ultrastructure of the meninges at the site of penetration of veins through the dura mater, with particular reference to Pacchionian granulations. Investigations in the rat and two species of New-World monkeys (Cebus apella, Callitrix jacchus). Cell Tissue Res.

[67] Liu X (2013). The superficial glia limitans of mouse and monkey brain and spinal cord. Anat Rec (Hoboken, N.J. : 2007).

[68] Hsu M (2019). Neuroinflammation-induced lymphangiogenesis near the cribriform plate contributes to drainage of CNS-derived antigens and immune cells. Nat Commun.

[69] Herisson F (2018). Direct vascular channels connect skull bone marrow and the brain surface enabling myeloid cell migration. Nat Neurosci.

[70] Yao H (2018). Leukaemia hijacks a neural mechanism to invade the central nervous system. Nature.

[71] Wolfe H (2018). Infiltrating macrophages contribute to age-related neuroinflammation in C57/BL6 mice. Mech Ageing Dev.

[72] Batterman KV (2021). T cells actively infiltrate the white matter of the aging monkey brain in relation to increased microglial reactivity and cognitive decline. Front Immunol.

[73] Stichel CC, Luebbert H (2007). Inflammatory processes in the aging mouse brain: participation of dendritic cells and T-cells. Neurobiol Aging.

[74] Honarpisheh P, Bryan RM, McCullough LD (2022). Aging Microbiota-gut-brain axis in stroke risk and outcome. Circ Res.

[75] Yanev P (2019). Impaired meningeal lymphatic vessel development worsens stroke outcome. J Cereb Blood Flow Metab.

[76] Esposito E (2019). Brain-to-cervical lymph node signaling after stroke. Nat Commun.

[77] Beuker C (2022). Stroke induces disease-specific myeloid cells in the brain parenchyma and pia. Nat Commun.

[78] Pu T (2019). Persistent malfunction of glymphatic and meningeal lymphatic drainage in a mouse model of subarachnoid hemorrhage. Exp Neurobiol.

[79] Rajan WD (2020). Defining molecular identity and fates of CNS-border associated macrophages after ischemic stroke in rodents and humans. Neurobiol Dis.

[80] Pedragosa J (2018). CNS-border associated macrophages respond to acute ischemic stroke attracting granulocytes and promoting vascular leakage. Acta Neuropathol Commun.

[81] Riew TR (2022). Infiltration of meningeal macrophages into the Virchow-Robin space after ischemic stroke in rats: Correlation with activated PDGFR-beta-positive adventitial fibroblasts. Front Mol Neurosci.

[82] Kosel S (1997). Long-lasting perivascular accumulation of major histocompatibility complex class II-positive lipophages in the spinal cord of stroke patients: possible relevance for the immune privilege of the brain. Acta Neuropathol.

[83] Xiang J (2017). The choroid plexus as a site of damage in hemorrhagic and ischemic stroke and its role in responding to injury. Fluids Barriers CNS.

[84] Li Y, Chen J, Chopp M (2002). Cell proliferation and differentiation from ependymal, subependymal and choroid plexus cells in response to stroke in rats. J Neurol Sci.

[85] Ennis SR, Keep RF (2006). The effects of cerebral ischemia on the rat choroid plexus. J Cereb Blood Flow Metab.

[86] Llovera G (2017). The choroid plexus is a key cerebral invasion route for T cells after stroke. Acta Neuropathol.

[87] Ge R (2017). Choroid plexus-cerebrospinal fluid route for monocyte-derived macrophages after stroke. J Neuroinflammation.

[88] Rayasam A (2022). Scavenger receptor CD36 governs recruitment of myeloid cells to the blood-CSF barrier after stroke in neonatal mice. J Neuroinflammation.

[89] van Rooijen N, Sanders A, van den Berg TK (1996). Apoptosis of macrophages induced by liposome-mediated intracellular delivery of clodronate and propamidine. J Immunol Methods.

[90] Polfliet MMJ (2001). A method for the selective depletion of perivascular and meningeal macrophages in the central nervous system. J Neuroimmunol.

[91] Wan H (2021). Role of perivascular and meningeal macrophages in outcome following experimental subarachnoid hemorrhage. J Cereb Blood Flow Metab.

[92] Coulibaly AP (2021). Neutrophil enzyme myeloperoxidase modulates neuronal response in a model of subarachnoid hemorrhage by venous injury. Stroke.

[93] Drieu A et al (2020) Alcohol exposure-induced neurovascular inflammatory priming impacts ischemic stroke and is linked with brain perivascular macrophages. JCI Insight 5(4):e129226 10.1172/jci.insight.129226 PMC7101143 31990687

[94] Strbian D (2006). Cerebral mast cells regulate early ischemic brain swelling and neutrophil accumulation. J Cereb Blood Flow Metab.

[95] Arac A (2014). Evidence that meningeal mast cells can worsen stroke pathology in mice. Am J Pathol.

[96] McKittrick CM, Lawrence CE, Carswell HV (2015). Mast cells promote blood brain barrier breakdown and neutrophil infiltration in a mouse model of focal cerebral ischemia. J Cereb Blood Flow Metab.

[97] Strbian D (2007). Mast cell blocking reduces brain edema and hematoma volume and improves outcome after experimental intracerebral hemorrhage. J Cereb Blood Flow Metab.

[98] Brea D (2021). Stroke affects intestinal immune cell trafficking to the central nervous system. Brain Behav Immun.

[99] Garcia JH (1994). Influx of leukocytes and platelets in an evolving brain infarct (Wistar rat). Am J Pathol.

[100] Enzmann G (2013). The neurovascular unit as a selective barrier to polymorphonuclear granulocyte (PMN) infiltration into the brain after ischemic injury. Acta Neuropathol.

[101] Pérez-de Puig I (2015). Neutrophil recruitment to the brain in mouse and human ischemic stroke. Acta Neuropathol.

[102] Möller K (2014). Sterile Inflammation after Permanent Distal MCA Occlusion in Hypertensive Rats. J Cereb Blood Flow Metab.

[103] Otxoa-de-Amezaga A (2019). Location of neutrophils in different compartments of the damaged mouse brain after severe ischemia/reperfusion. Stroke.

[104] Hawkes CA, McLaurin J (2009). Selective targeting of perivascular macrophages for clearance of beta-amyloid in cerebral amyloid angiopathy. Proc Natl Acad Sci U S A.

[105] Park L (2017). Brain perivascular macrophages initiate the neurovascular dysfunction of Alzheimer Aβ peptides. Circ Res.

[106] Rohde D (2022). Bone marrow endothelial dysfunction promotes myeloid cell expansion in cardiovascular disease. Nat Cardiovasc Res.

[107] Kaiser D (2014). Spontaneous white matter damage, cognitive decline and neuroinflammation in middle-aged hypertensive rats: an animal model of early-stage cerebral small vessel disease. Acta Neuropathol Commun.

[108] Iyonaga T (2020). Brain perivascular macrophages contribute to the development of hypertension in stroke-prone spontaneously hypertensive rats via sympathetic activation. Hypertens Res.

[109] Yu Y (2010). Brain perivascular macrophages and the sympathetic response to inflammation in rats after myocardial infarction. Hypertension.

[110] Santisteban MM (2020). Endothelium-Macrophage Crosstalk Mediates Blood-Brain Barrier Dysfunction in Hypertension. Hypertension.

[111] Faraco G (2016). Perivascular macrophages mediate the neurovascular and cognitive dysfunction associated with hypertension. J Clin Investig.

[112] Santisteban MM et al (2022) Meningeal IL-17 producing T cells mediate cognitive impairment in salt-sensitive hypertension. bioRxiv. 10.1101/2022.09.05.506398 10.1038/s41593-023-01497-z PMC10999222 38049579

[113] Hartlehnert M (2021). Bcl6 controls meningeal Th17-B cell interaction in murine neuroinflammation. Proc Natl Acad Sci U S A.

[114] Bolte AC (2023). The meningeal transcriptional response to traumatic brain injury and aging. Elife.

